# Monkeypox: Another Sexually Transmitted Infection?

**DOI:** 10.3390/pathogens11070713

**Published:** 2022-06-21

**Authors:** Alfonso J. Rodriguez-Morales, Gustavo Lopardo

**Affiliations:** 1 Grupo de Investigación Biomedicina, Faculty of Medicine, Fundacion Universitaria Autonoma de las Americas, Pereira 660003, Colombia; 2 Clinical Epidemiology and Biostatistics, Universidad Cientifica del Sur, Lima 15067, Peru; 3 Faculty of Medicine, Universidad de Buenos Aires, Buenos Aires C1120AAQ, Argentina; glopardo@intramed.net

Monkeypox virus is a zoonotic DNA virus (Poxviridae family), identified in 1958 in Asian monkeys (mostly *Macaca fascicularis*) in a polio vaccine research animal facility in Copenhagen, Denmark. In 1970 also in humans in the Democratic Republic of the Congo, a 9-month-old boy in a region where smallpox had been eliminated in 1968 [1,2]. It was considered endemic in West and Central African countries, with two clades, a milder and a more pathogenic one, respectively, marked geographically by Cameroon, which has both clades in the middle of them [3]. The first outbreak outside Africa occurred in 2003 due to imported wild rodents for pet use in the United States of America (USA) children from 6 states (Wisconsin, and Indiana, among others). That outbreak was exclusively animal-to-human transmission [4]. 

Since 2018, sporadic imported cases have been reported in the United Kingdom (UK), Singapore and the USA. But, recently, in 2022, with the concurrent COVID-19 pandemic [5], an ongoing outbreak in the UK and other European and non-European countries has also been reported, with 2677 cases confirmed up to 21 June 2022 [6]. This multi-country outbreak is occurring with the sustainable human-to-human transmission among non-travellers, to a large extent amongst men who have sex with men (MSM) [7,8]. Then, concern has been raised due to the suspicion that monkeypox has been transmitted sexually [9]. Some of those cases occur in MSM living with HIV, and one recently syphilis coinfection has been reported in the Czech Republic [9]. Also, viral detection in seminal fluid, genital and rectal lesions, and faeces of four MSM in Italy was recently associated with the 2022 outbreak. The monkeypox virus was positive in three patients from their seminal fluid, with a quantification cycle ranging from 27 to 30, low for viral isolation [10]. Although it is clear that studies are necessary to confirm global suspicion, current findings support the hypothesis that monkeypox can be sexually transmitted. While these findings cannot indicate definitive evidence of infectivity, they demonstrate viral shedding, and its efficiency for transmission cannot yet be ruled out [10].

That poses a risk deserving education, awareness and prevention in the sexually active population, mainly, but not only, among the LGBTI community, as recently suggested by the European Centers for Disease Control (ECDC) [11]. ECDC indicated that transmission probability is considered high among persons with multiple sexual partners, including MSM [11]. 

Initially considered only zoonotic, the virus has shown potential for interhuman transmission via close contact with lesions, body fluids, respiratory droplets and contaminated materials for decades [9]. However, it would also be of concern its sexual transmission [9,11]. These considerations would also be important in assessing HIV and other STI patients presenting with a vesicular-papular rash after a prodrome of fever of 3–5 days, with lymphadenopathy [12], including also genital ulcers, especially after visiting cities with confirmed cases [6]. At least in 52 of the confirmed cases, genital ulcers had been reported. 

In studies, preliminary data suggest that risk factors for monkeypox include being a young male, having sex with other men, engaging in risky behaviours and activities, including condomless sex, seropositivity HIV positivity, and a history of previous STI, including syphilis [13]. Multiple partners, anonymous partners, condomless sex, and substance use are all associated with an increased risk of HIV infection. Notably, other STIs also might significantly increase the risk for HIV infection. An estimated 10% of new HIV infections were attributable to the chlamydial or gonococcal disease. Recently published reports have described cases of Monkeypox with genital lesions, a factor associated with an increased risk of acquisition of HIV infection [14,15,16].

Zoonotic disease programs would also need interaction and integrated strategies with sexual health and sexually transmitted infections programs to control this potentially new sexually transmitted infection effectively.

As discussed in this introductory Editorial, there are multiple implications of discovering an old disease and its aetiology, now with the involvement of sexual transmission. That is not new. Similarly, during the 2015–2017 Zika epidemic (then declared a Public Health Emergency of International Concern by the World Health Organization), the sexual transmission of that arbovirus also occurred [17,18,19]. In the Special Issue “Human Monkeypox: An Emerging Sexually Transmitted Infection?”, multiple articles are expected to address this problem and suggest solutions.

Finally, scientific associations, such as the International Society for Infectious Diseases (ISID), the International Society for Antimicrobial Chemotherapy (ISAC), the Infectious Diseases Society of the Americas (IDSA), the HIV Medicine Association (HMA), the British HIV Association (BHA), the European Society for Clinical Microbiology and Infectious Diseases (ESCMID), the Pan-American Infectious Diseases Association (API), or national societies, e.g., in Colombia, the Association of Infectious Diseases (ACIN), or in Argentina, the Argentinian Society for Infectious Diseases (SADI), should lead great efforts to deal with this emerging problem during the midst of a pandemic of COVID-19 that has not ceased yet, which threatens to increase the already established problem of STIs in the world, that increase in the number of associated pathogens [20]. 

## Data Availability

Not applicable.
